# Rapid ATF4 Depletion Resets Synaptic Responsiveness after cLTP

**DOI:** 10.1523/ENEURO.0239-20.2021

**Published:** 2021-06-02

**Authors:** Fatou Amar, Carlo Corona, Johanna Husson, Jin Liu, Michael Shelanski, Lloyd Greene

**Affiliations:** 1 Department of Pathology and Cell Biology, Columbia University Medical Center, Vagelos College of Physicians and Surgeons, Columbia University, New York, New York 10032; 2 The Taub Institute for Research on Alzheimer’s Disease and the Aging Brain, Columbia University, New York, New York 10032

**Keywords:** AMPA receptor, ATF4, LTP, resetting synaptic activity, synaptic plasticity

## Abstract

Activating transcription factor 4 [ATF4 (also called CREB2)], in addition to its well studied role in stress responses, is proposed to play important physiologic functions in regulating learning and memory. However, the nature of these functions has not been well defined and is subject to apparently disparate views. Here, we provide evidence that ATF4 is a regulator of excitability during synaptic plasticity. We evaluated the role of ATF4 in mature hippocampal cultures subjected to a brief chemically induced LTP (cLTP) protocol that results in changes in mEPSC properties and synaptic AMPA receptor density 1 h later, with return to baseline by 24 h. We find that ATF4 protein, but not its mRNA, is rapidly depleted by ∼50% in response to cLTP induction via NMDA receptor activation. Depletion is detectable in dendrites within 15 min and in cell bodies by 1 h, and returns to baseline by 8 h. Such changes correlate with a parallel depletion of phospho-eIF2a, suggesting that ATF4 loss is driven by decreased translation. To probe the physiologic role of cLTP-induced ATF4 depletion, we constitutively overexpressed the protein. Reversing ATF4 depletion by overexpression blocked the recovery of synaptic activity and AMPA receptor density to baseline values that would otherwise occur 24 h after cLTP induction. This reversal was not reproduced by a transcriptionally inactive ATF4 mutant. These findings support the role of ATF4 as a required element in resetting baseline synaptic responsiveness after cLTP.

## Significance Statement

The mechanisms by which synaptic responsiveness is reset after LTP are not well understood. Resetting avoids LTP “saturation” and uncontrolled feedforward potentiation and may play a part in synaptic “scaling.” In the work reported here, we have found that the activating transcription factor 4 (ATF4) is translationally downregulated following chemical LTP induction and acts as a regulator of long-term synaptic plasticity, resetting the synapse to the depotentiated state. Our findings may serve to begin to reconcile the conflicting views regarding the role of ATF4 in synaptic plasticity and further illuminate the role of ATF4 in learning and memory.

## Introduction

Although often considered in pathologic contexts in the brain as a responder to stress (Pitale et al., 2017), much evidence has pointed to roles for activating transcription factor 4 [ATF4 (also called CREB2)] in physiologic neuronal function (Bartsch et al., 1995; Costa-Mattioli et al., 2007; Liu et al., 2014; Hu et al., 2015; Pasini et al., 2015). The features of ATF4 make it especially suitable for a potential role in synaptic plasticity. It is detectably expressed in neurons, and in particular in neuronal processes, and undergoes retrograde transport to cell bodies and nuclei, where it regulates a variety of genes relevant to neuronal function (Lai et al., 2008; Sun et al., 2013; Baleriola et al., 2014; Pasini et al., 2016; Liu et al., 2018). ATF4 is also subject to rapidly regulated expression (Ameri and Harris, 2008). The latter property arises from the fast turnover and regulated translation of ATF4 via eukaryotic translation initiation factor 2a (eIF2a). When eIF2a is in a nonphosphorylated state, it promotes global translation, but suppresses translation of a subset of mRNAs including the mRNA encoding ATF4 (Harding et al., 2000). Conversely, when eIF2a is phosphorylated (p) by a defined set of kinases, global translation is reduced and translation of the subset of messages, including that encoding ATF4, is selectively enhanced (Harding et al., 2000; Ameri and Harris, 2008).

While there is general agreement that ATF4 appears to play an important role in synaptic plasticity as well as in learning and memory, there are divergent views about whether such activities are positive or negative (Bartsch et al., 1995; Chen et al., 2003; Costa-Mattioli et al., 2005, 2007; Trinh et al., 2012; ILL-Raga et al., 2013; Liu et al., 2014; Hu et al., 2015; Pasini et al., 2015). Such disparate findings may, in part, reflect that much of the evidence is based on indirect control of ATF4 expression/activity by manipulating eIF2a phosphorylation levels or by blockade with dominant-negative inhibitors that might also inhibit other members of the b-ZIP transcription factor family, to which ATF4 belongs. In past work, to more directly gauge the physiologic role ATF4 in brain, we have directly manipulated its expression in hippocampal neurons in culture and *in vivo*, and reported that chronic ATF4 downregulation or depletion decreases mushroom spine density, reduces excitatory synapses, produces deficits in long-term spatial memory and behavioral flexibility, impairs both long-term potentiation (LTP) and long-term depression as well as glutamatergic function, and diminishes GABA_B_ receptor trafficking (Liu et al., 2014; Pasini et al., 2015; Corona et al., 2018). Strikingly, while ATF4 overexpression reverses such parameters, it does not elevate them above baseline.

Here, to further understand the role of ATF4 in neuronal plasticity, we have examined both its regulation and function in a form of LTP, a cellular mechanism associated with learning and memory (Bliss and Collingridge, 1993; Chen and Tonegawa, 1997; Malenka and Nicoll, 1999; Pittenger and Kandel, 2003; Dudai, 2004). Although LTP must last for several hours to promote long-term changes in memory, it also must be capable of “resetting” to unstimulated levels for a variety of reasons, including avoidance of saturation and promotion of additional rounds of plasticity (Moser et al., 1998), providing synaptic scaling (Turrigiano, 2008), and forestalling excessive excitatory activity that could lead to seizures (Bliss and Cooke, 2011) or even neuronal damage (McEachern and Shaw, 1999). In this context, we report that chemically induced LTP (cLTP) evoked by brief exposure to glutamate leads to a rapid decrease of ATF4 protein levels in hippocampal neurons and that reversal of such depletion by ATF4 overexpression blocks long-term resetting of the LTP mechanism back to baseline. These findings suggest that ATF4 acts as a feedback regulator of synaptic plasticity associated with LTP.

## Materials and Methods

### DNA constructs

Lentiviral constructs were generated as previously described (Liu et al., 2014).

### Lentivirus preparation

For *in vitro* experiments, the second-generation packaging system (which generates replication-deficient lentivirus) was used for all experiments to prepare lentivirus (Zufferey et al., 1997). Packaging vectors psPAX2 and pMD2.G were obtained from Addgene. In summary, lentiviral constructs for overexpression were cotransfected with the packaging vectors into HEK293T cells with calcium phosphate. Supernatants from HEK293T cells infected with virus were collected 24 and 48 h after transfection. After centrifugation at 1000 rpm for 10 min, the supernatants were passed through a 0.45 μm PVDF filter unit (Nalgene) then concentrated 20–30× by centrifugation in an Amicon Ultra Centrifugal Filter (model 100 K, Millipore) following the manufacturer instructions. Viruses were aliquoted and stored at −80°C. Viral titers ranged from 1 to 5 × 10^6^ infectious units/μl.

### Cell culture and infection

Primary hippocampal cultures were prepared as previously described (Corona et al., 2018; Liu et al., 2018). Briefly, hippocampi from E18 rat embryos were dissected out, dissociated, and plated on poly-d-lysine-coated plates or coverslips (Sigma-Aldrich) in 12-well plates at a density of 3 × 10^5^/well. For cell staining experiments, neurons were cultured at a low density (3 × 10^4^/well) on cover glasses and maintained in conditioned medium (from regular density cultures). Neurons were maintained in Invitrogen Neurobasal medium (Thermo Fisher Scientific) supplemented with 2% B-27 medium (Thermo Fisher Scientific) and 0.5 mm glutamine (Thermo Fisher Scientific). Half of the culture medium was changed every 3 d after plating. For overexpression experiments, lentiviruses were added to the cultures on days 20–21 *in vitro*, and the cultures were used 1 d later for electrophysiology or fixed for staining.

### Chemical induction of long-term potentiation

Long-term potentiation was chemically induced on mature hippocampal cultures as previously described (Malgaroli and Tsien, 1992). Briefly, mature hippocampal cultures were washed three times in prewarmed Tyrode’s buffer (119 mm NaCl, 5 mm KCl, 20 mm HEPES, 30 mm glucose, 30 mm sucrose, 2 mm CaCl_2_, pH 7.3) 5 min before induction. Then cultures were quickly washed three times with Tyrode’s buffer containing 50 μm l-glutamate and 1 μm glycine, and then incubated with the glutamate-containing buffer for 30 s. After the 30 s incubation cultures were washed three times in buffer without glutamate or glycine. Vehicle-treated cultures were similarly treated with Tyrode’s buffer only.

### Antibodies and Western immunoblotting

For Western immunoblotting analysis, treated neurons were collected in 1× LDS Loading Buffer (Thermo Fisher Scientific) and boiled for 10 min. Proteins were separated by electrophoresis in 10% NuPAGE gels (Thermo Fisher Scientific). To better detect ATF4, the gels were run a longer time (80–100 min) to separate ATF4 from a closely migrating nonspecific band. The following primary antibodies were used: rabbit monoclonal anti-ATF4 (1:1000), rabbit anti-eIF2a (1:1000), rabbit anti-p-eIF2a (1:1000), anti-CREB (1:1000), and anti-pCREB (1:2000), all from Cell Signaling Technology; and mouse anti-GAPDH (1:2000) from Imgenex. For secondary antibody, HRP-conjugated anti-rabbit and anti-mouse secondary antibody (1:5000; Thermo Fisher Scientific) was used. The ATF4 antibodies were validated by assessment in cultures in which ATF4 had been knocked down with lentiviral-delivered short hairpin ATF4 (shATF4; Corona et al., 2018).

### Immunofluorescence labeling

At 15 min, 1 h, or 24 h post-cLTP treatment, the cells were fixed with 4% paraformaldehyde for 10 min. For immunostaining of excitatory synaptic puncta, double labeling of surface GluA1 and intracellular PSD-95 was conducted. Neurons were first labeled with a rabbit monoclonal antibody directed against the N-terminal extracellular domain of the GluA1 receptor (1:300; Cell Signaling Technology) and a secondary anti-rabbit antibody Alexa Fluor 568 (1:500; Thermo Fisher Scientific) under nonpermeabilizing conditions. Cells were then labeled with a mouse monoclonal anti-PSD-95 (1:150; Thermo Fisher Scientific) and anti-mouse secondary antibody Alexa Fluor 488 (1:300; Thermo Fisher Scientific), or, for infected neurons, Alexa Fluor 680 (1:300; Thermo Fisher Scientific) after permeabilization of the cellular membranes with PBS + 0.25% Triton X-100 for 2× 30 min. For ATF4 labeling, after fixation and permeabilization, cells were probed with ATF4 antibody (1:200; Cell Signaling Technology rabbit monoclonal antibody D4B8, catalog #11815) and then secondary anti-rabbit antibody Alexa Fluor 568 (1:500;Thermo Fisher Scientific). DAPI (1:8000) was finally added for 8 min to stain nuclei. For β-III-tubulin, labeling was conducted with mouse monoclonal antibody TU-20 (1:300; catalog #NB-600–1018, Novus Biologicals), then a secondary anti-mouse antibody, Alexa Fluor 647 (1:500; Thermo Fisher Scientific). The coverslips were mounted on slides with the mounting agent Prolong Gold (Thermo Fisher Scientific).

### Image acquisition and quantification

Images were acquired with a confocal microscope (catalog #LSM800, Zeiss) using a 40× oil-objective and 2.4× zoom with sequential acquisition at 1024 × 1024 pixels. Each acquisition was a *z*-stack series of 17–19 images per channel. The lasers used were 405 nm for DAPI, 488 nm for AF488, 561 nm for AF568, and 640 nm for AF680. The interval was 0.2 μm between images. The confocal microscope settings were the same for the acquisition of images for the control and treatment conditions within one experiment and across experiments using the same channels. Experiments using the three channels AF568, AF488, and DAPI share a set of parameters, and experiments using the four channels AF568, AF488, AF680, and DAPI share another set of parameters. To select a neuron for analysis, the visual field was moved blindly to a random site on the coverslip in the DAPI channel. A neuron with a healthy nucleus (round-shaped DAPI staining) was selected randomly and imaged. Between 7 and 13 neurons were imaged for each condition of an experiment. Evaluation of apoptotic nuclei after DAPI staining was conducted on nonsaturated images of randomly chosen fields.

Images were analyzed with ImageJ software. The signal was normalized for background and area using the formula IntDen – (area * background intensity). The stack of images was grouped in a single image by averaging intensity. Brightness and contrast parameters were adjusted to eliminate background and enhance visualization of ATF4, GluA1, and PSD-95 signal, and were kept identical across the control and treatment conditions within each experiment. For puncta analysis, all quantifications were performed within rectangles of identical size (20 × 5 μm) drawn along three different dendrites for each neuron. Quantification of the density of surface GluA1 clusters, intracellular PSD-95 clusters, and yellow clusters of colocalization was performed manually and blindly. For ATF4 analysis, total ATF4 signal was quantified using the software ImageJ. Signal for ATF4 was measured from each image field and plotted for total ATF4 levels normalized to PSD-95 with same number of ATF4-positive cells for both vehicle-treated and cLTP-treated cultures. For cell body ATF4 quantification, ATF4 fluorescent signal was measured in each cell body and normalized to cell body area in each field with the same number of cells in each condition. ATF4 signal in processes was obtained by subtracting cell body ATF4 signal in all the cells in each field from total AFT4 signal.

### Quantitative real-time PCR

To assess the level of endogenous ATF4 mRNA in cultured neurons, total RNA was isolated from rat primary hippocampal cultures at 1 and 24 h after cLTP induction or control by using RNeasy Mini Kit (Qiagen). RNA concentration and purity were determined using a NanoDrop 8000 (Thermo Fisher Scientific). Reverse transcription was performed by using the First-strand cDNA Synthesis Kit (Origene) following the manufacturer’s instructions. Reaction mixtures were diluted fivefold and subjected to quantitative real-time PCR amplification (Eppendorf) using FastStart SYBR Green Master mix (Roche). The following primers were used: ATF4: forward 5′-ATGCCAGATGAGCTCTTGACCAC-3′ and reverse 5′-GTCATTGTCAGAGGGAGTGTCTTC-3′; and 18S forward 5′-TTGATTAAGTCCCTGCCCTTTGT-3′ and reverse 5′-CGATCCGAGGGCCTCACTA-3′.

Relative product quantities for each transcript were performed in triplicate, normalized to 18S mRNA as an endogenous control, and determined using the comparative C_T_ method.

### Electrophysiology

Primary hippocampal neurons [20–21 d *in vitro* (DIV), 1–2 d after lentiviral infection when indicated, and 30 min and 24 h following cLTP induction] were used for tight-seal conventional whole-cell patch clamp. All currents were recorded from pyramidal-like neurons based on the their large (∼15 μm) triangular-shaped somas. cLTP was induced as described above and at the indicated times after induction, coverslips were placed in a recording chamber with a HEPES-buffered bath solution containing the following (in mm): 119 NaCl, 5 KCl, 20 HEPES, 30 glucose, 2 CaCl_2_, 2 MgCl_2_. The pH and osmolarity of the bath solution were adjusted to 7.3 and 330 mOsm/L, respectively. For miniature EPSC (mEPSC) recordings, glass pipettes were filled with the following intracellular electrode solution, pH 7.3 (285 mOsm/L) containing the following (in mm): 130 K-gluconate, 10 KCl, 10 HEPES, 1 MgCl_2_, 0.06 CaCl_2_, 0.1 EGTA, 3 MgATP, and 0.3 Na_2_GTP, and typically registered 4–8 mΩ pipette resistances. Furthermore, 1 μm TTX and 100 μm picrotoxin were continuously perfused during the experiment. All neurons were recorded at −70 mV for 10 min, and a 5 mV hyperpolarizing test pulse was applied periodically during recordings to ensure that the access resistance did not change significantly and was <25 mΩ. If not, the recordings were discarded. Signals were filtered at 2 kHz, digitized at 10 kHz, stored, and analyzed offline using MiniAnalysis Software (version 6.0.7, Synaptosoft). The threshold for event detection was set at 5 pA. Recordings were performed at room temperature under constant perfusion (2 ml/min) and acquired using Clampex software and a microamplifier (MultiClamp 700B, Molecular Devices).

### Statistical analysis

Data are shown as mean values ± SEM. Comparison between two groups was performed with a two-tailed paired Student’s *t* test. Once the direction of change was established, and subsequent comparisons were performed with a one-tailed *t* test as indicated. Comparison between multiple groups and comparison of curves were performed using one-way ANOVA, followed by Bonferroni *post hoc* test when applicable. Statistical significance was set for *p* < 0.05.

## Results

### Time-dependent downregulation of ATF4 following cLTP

**Figure 1.**
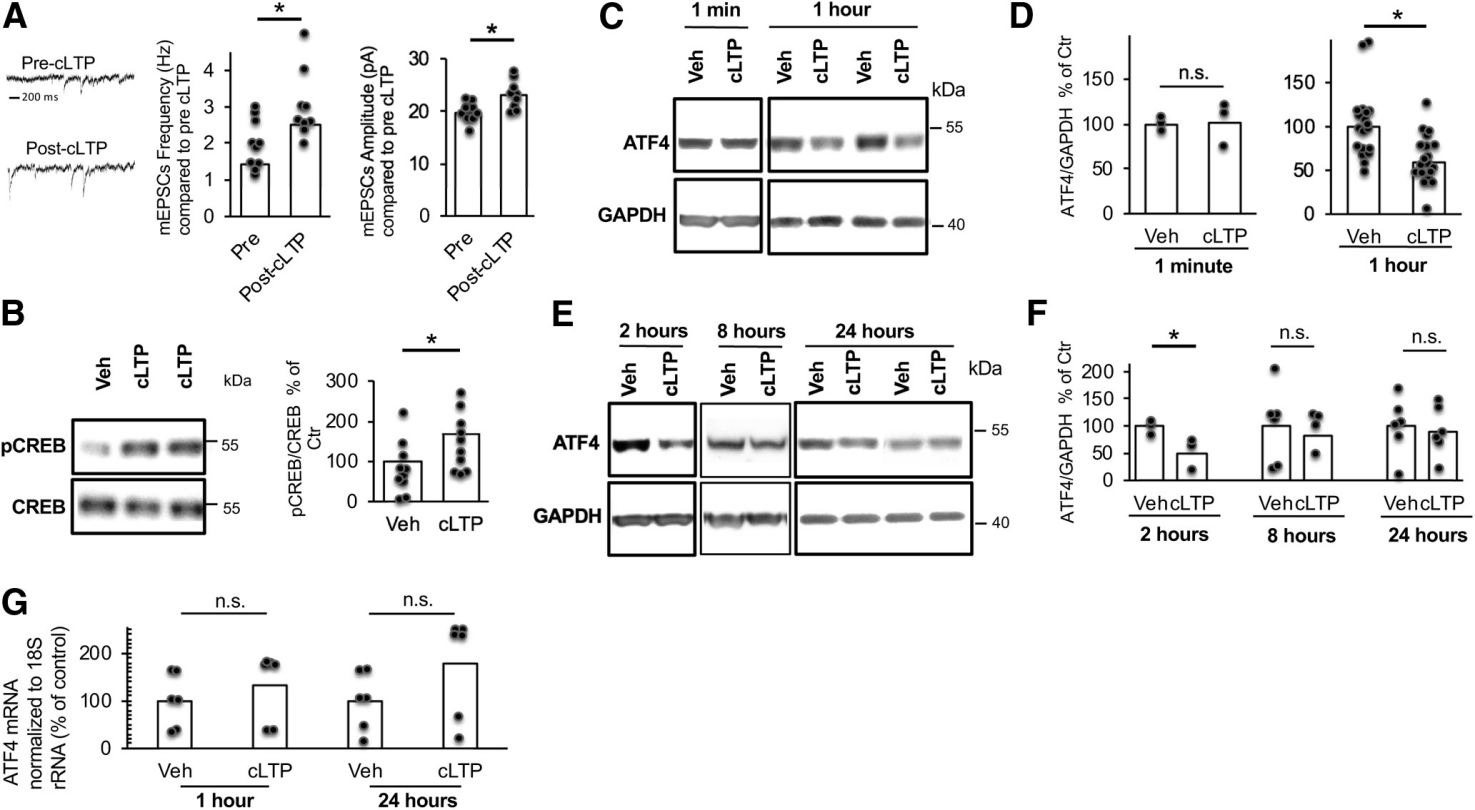
Time-dependent downregulation of ATF4 following cLTP induction. ***A***, Bar graphs represent the frequency (left) and the amplitude (right) of mEPSCs recorded from 21 DIV hippocampal neurons before or 1 h after cLTP induction (*N* = 9–10/condition). ***B***, Representative WB of p-CREB and total CREB proteins in DIV 21 hippocampal cultures 1 h following cLTP induction or vehicle treatment with quantification of *N* = 10/condition. ***C***, Representative WB of ATF4 and GAPDH in DIV 21 hippocampal cultures at 1 min and 1 h post-cLTP induction or vehicle treatment. ***D***, Quantification of multiple experiments as depicted in ***C*** (*N* = 20/condition). ***E***, Representative WB of ATF4 and GAPDH in DIV 21 hippocampal cultures at 2, 8, and 24 h post-LTP induction or vehicle treatment. ***F***, Quantification of multiple experiments as depicted in ***E***, 2 h (*N* = 3/condition), 8 h (*N* = 5–6/condition), and 24 h (*N* = 6/condition) post-cLTP. ***G***, Q-PCR was conducted to quantify ATF4 mRNA and 18S rRNA levels in DIV 21 hippocampal neurons at 1 and 24 h following cLTP induction or vehicle treatment. *N* = 6/condition. For this and all following figures: *p < 0.05 (see text for further details of statistics). Values of bars are means. n.s., Not significant. Dots show individual data points.

**Figure 2.**
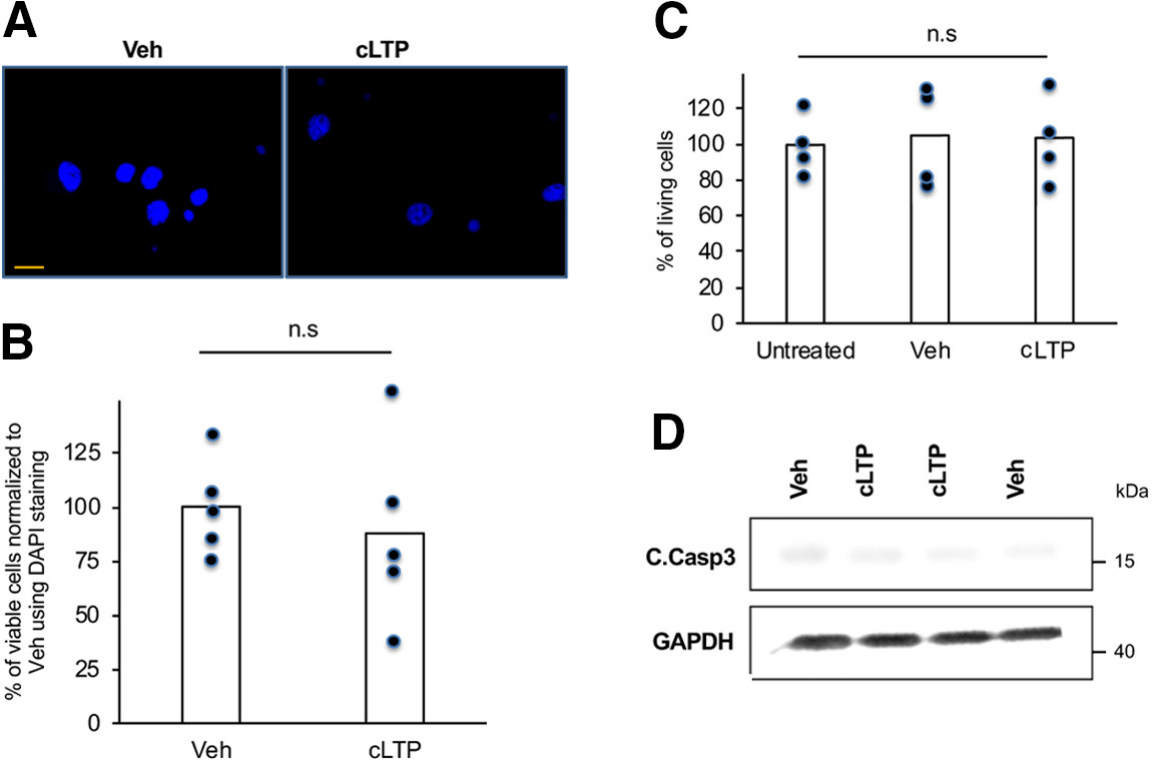
The cLTP protocol used here does not appear to induce toxicity in hippocampal cultures. ***A***, Fluorescent staining of DAPI (blue) in DIV 21 hippocampal cultures at 24 h following cLTP induction or vehicle treatment. Scale bar, 50 μm. ***B***, Quantification of percentages of cells with condensed chromatin, condensed nuclei, or nuclear fragmentation under indicated conditions. *N* = 5 fields/condition. ***C***, cLTP does not significantly affect cell numbers in hippocampal cultures at 24 h. Cultures were lysed at 24 h following cLTP or exposure to vehicle, and the number of viable cells was assessed by examining condensed nuclei. Values indicate the percentages of cells with intact nuclei under indicated conditions. *N* = 4 fields/condition. ***D***, Representative WB of cleaved caspase-3 (C. Casp3) and GAPDH in DIV 21 hippocampal cultures at 24 h following cLTP induction or vehicle treatment.

Our studies and the studies of others have implicated ATF4, using both direct and indirect manipulations, as a regulator of neuronal plasticity (Bartsch et al., 1995; Costa-Mattioli et al., 2007; Liu et al., 2014; Pasini et al., 2015). If ATF4 is to participate in plasticity, one potential mechanism is modulation of its levels in response to changes in synaptic activity. To test this, we examined whether ATF4 expression is affected by activity changes that induce LTP, a physiologic condition associated with learning and memory. We additionally asked whether such changes in turn affect neuronal plasticity. For this purpose, we turned to mature rat hippocampal cultures in which LTP is induced chemically (cLTP) by the well described protocol of 30 s exposure to 50 μm l-glutamate/1 μm glycine in Mg^2+^ free Tyrode’s buffer (Malgaroli and Tsien, 1992). This approach has the advantages of global synaptic activation that promotes ready detection of cellular and molecular changes in cultures that can be maintained for extended periods beyond cLTP induction. As previously described, cLTP induction is characterized by increases in the frequency and amplitude of spontaneous mEPSCs and elevation of CREB phosphorylation (Malgaroli and Tsien, 1992). Consistent with this, we found a 1.8-fold increase in mEPSC frequency (*t*_(17)_ = 2.82, *p* = 0.012, two-tailed *t* test) and an almost 20% increase in mEPSC amplitude (*t*_(17)_ = 3.23, *p* = 0.0049, two-tailed *t* test) within 30 min of cLTP induction (Fig. 1*A*). Additionally, compared with cultures treated with vehicle, the levels of phosphorylated CREB, analyzed by Western immunoblotting (WB), increased significantly (*t*_(18)_ = 2.09, *p* = 0.026, one-tailed *t* test) in cultures 1 h post-cLTP induction (Fig. 1*B*). To detect possible toxicity caused by the cLTP conditions, we visually monitored the cultures, assessed total viable cell numbers, and conducted DAPI staining to identify cells with condensed chromatin, condensed nuclei, or nuclear fragmentation at up to 24 h post-treatment. Compared with the vehicle-treated condition, cLTP treatment did not significantly promote DNA damage or affect the numbers of viable cells (Fig. 2*A–C*). There was also no evident effect on the appearance or morphology of the cultured neurons. Additionally, the levels of cleaved caspase 3, which increase robustly in dying cultured hippocampal neurons (Lefort et al., 2012), were unchanged in the cultures at 24 h post-cLTP induction (Fig. 2*D*).

We next examined ATF4 protein levels in the cultures at various times after cLTP induction by WB using a previously validated antiserum (Corona et al., 2018). Compared with vehicle treatment, cells subjected to cLTP showed time-dependent changes in ATF4 protein levels (Fig. 1). Within 1 h of cLTP induction, we observed a significant decrease (*t*_(38)_ = 3.82, *p* = 0.0005, two-tailed *t* test) in ATF4 expression, and this persisted for 2 h (*t*_(4)_ = 2.41, *p* = 0.037, one- tailed *t* test; Fig. 1*C–F*) to a level 40–50% lower than in control cultures. At 8 h post-cLTP, ATF4 protein levels were no longer significantly different compared with vehicle (*t*_(9)_ =0.49, *p* = 0.32, one-tailed *t* test; Fig. 1*E*,*F*), and by 24 h post-cLTP, ATF4 protein remained at levels present in unstimulated cultures (*t*_(10)_ = 0.33, *p* = 0.37, one-tailed *t* test; Fig. 1*E*,*F*). In contrast to protein, the assessment of ATF4 mRNA levels revealed no significant decreases at 1 or 24 h after cLTP induction (Fig. 1*G*; 1 h, *t*_(10)_ = 0.8, *p* = 0.44, two-tailed *t* test; 24 h, *t*_(10)_ = 1.53, *p* = 0.16, two-tailed *t* test). These findings thus indicate that cLTP induction in hippocampal cultures causes a rapid decrease in ATF4 protein levels that reverses by 8–24 h, and that this is not reflected by changes in ATF4 mRNA.

To determine whether the observed effects of cLTP on ATF4 expression occurred within neurons and, if so, whether these might be localized, we next conducted immunofluorescent labeling of ATF4. To validate the antibody used for this purpose, we immunostained cultures with or without shRNA ATF4 knockdown with a previously described lentivirally delivered shRNA (Liu et al., 2014; Corona et al., 2018). Infection with lentivirus expressing shATF4 resulted in a pronounced loss of signal within both cell bodies and processes compared with cultures infected with control virus (Extended Data Fig. 3-1).

**Figure 3.**
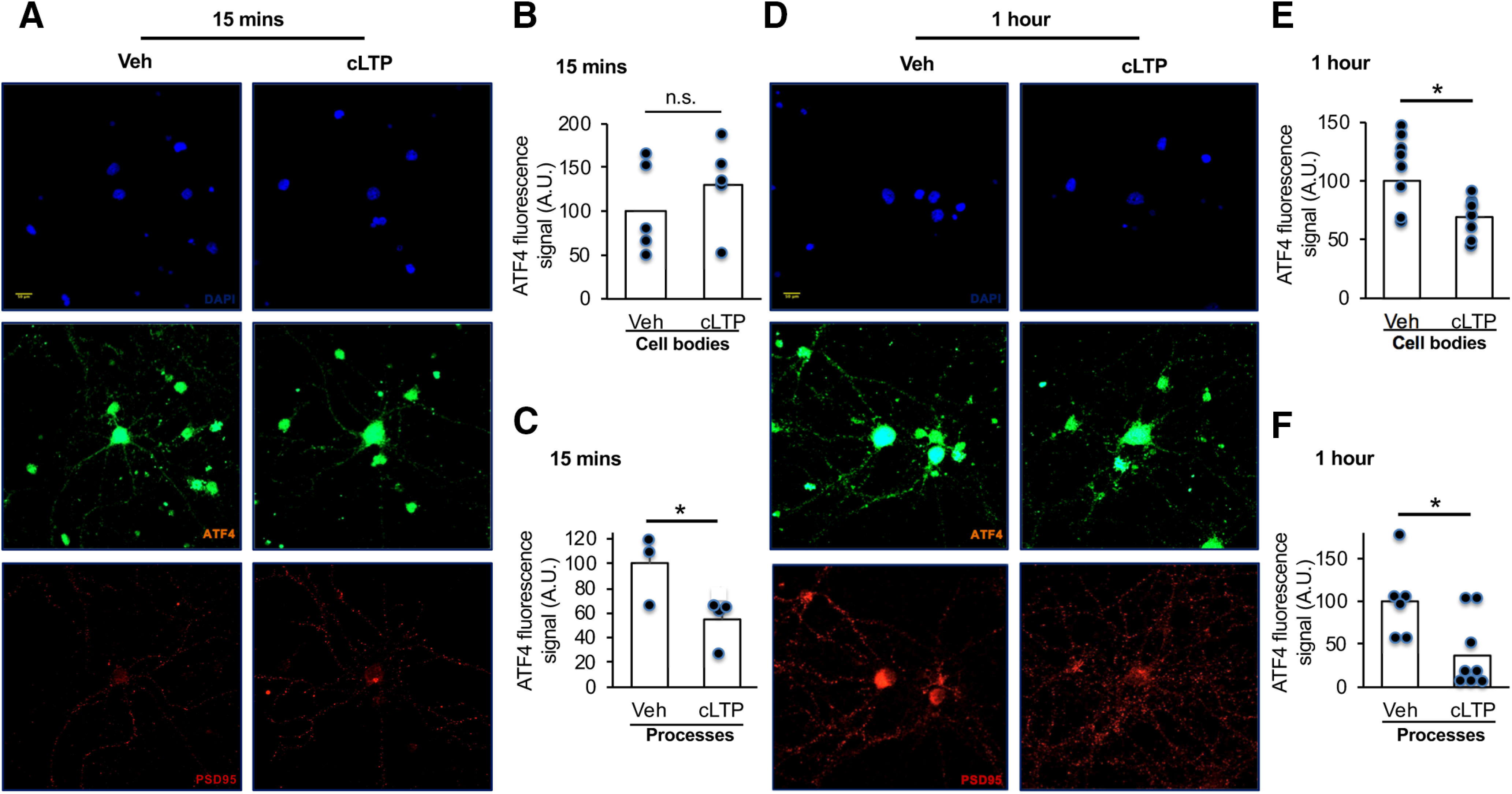
LTP-induced ATF4 downregulation initiates in processes. ***A***, Immunofluorescent staining of ATF4 (green), PSD-95 (red), and DAPI (blue) in DIV 21 hippocampal cultures 15 min following cLTP induction or vehicle treatment. ***B***, Quantification of ATF4 signals in cell bodies (normalized to PSD-95 fluorescence) in images as represented in ***A***. *N* = 5 images/condition. ***C***, Quantification of ATF4 signals in processes (normalized to PSD-95 fluorescence) in images as represented in ***A***. *N* = 3–4 images/condition. ***D***, Immunofluorescent staining of ATF4 (green), PSD-95 (red), and DAPI (blue) in DIV 21 hippocampal cultures 1 h following cLTP induction or vehicle treatment. ***E***, Quantification of ATF4 signals in cell body (normalized to PSD-95 fluorescence) in images as represented in ***D***. *N* = 8–9 images/condition. ***F***, Quantification of ATF4 signals in processes (normalized to PSD-95 fluorescence) in images as represented in ***D***. *N* = 6–9 images/condition. A.U., Arbitrary units. Scale bar, 50 μm. Data validating the specificity of the antibody used for these experiments are presented in Extended Data Figure 3-1.

10.1523/ENEURO.0239-20.2021.f3-1 Figure 3-1 Validation of the antibody used for immunofluorescent staining of cultured hippocampal neurons. DIV 7 cultures were infected with lentivirus expressing either GFP (control) or GFP + shATF4, and fixed and stained on DIV 21. For ATF4 labeling, following fixing, cells were probed as described in Materials and Methods with ATF4 antibody (1:300; Cell Signaling Technology), rabbit monoclonal antibody D4B8 (catalog #11815, Thermo Fisher Scientific), and then a secondary anti-rabbit antibody, Alexa Fluor 568 (1:500; Thermo Fisher Scientific) after permeabilization. For β-III-tubulin, labeling was conducted with mouse monoclonal antibody TU-20 (1:300; catalog #NB-600-1018, Novus Biologicals), then a secondary anti-mouse antibody, Alexa Fluor 647 (1:500; Thermo Fisher Scientific). Top, Staining as imaged on an upright fluorescent microscope. Bottom, Staining as imaged by confocal microscopy (merged *z*-stack). Download Figure 3-1, TIF file.

As previously reported (Liu et al., 2014), ATF4 within control cultures was present in neurons and was distributed both in processes (in a punctate pattern) and in cell bodies. At 15 min post-cLTP, while cell body signals for ATF4 were unchanged (*t*_(8)_ = 0.88, *p* = 0.2, one-tailed *t* test, *n* = 5 images/condition) compared with the vehicle, the ATF4 signal in processes was significantly decreased (*t*_(5)_ = 2.5, *p* = 0.027, one-tailed *t* test, *n* = 5 images/condition; Fig. 3*A–C*). By 1 h post-cLTP, the ATF4 signal in cell bodies was now significantly decreased (*t*_(15)_ = 1.78, *p* = 0.047, one-tailed *t* test, *n* = 5 images/condition) and in processes had fallen by an average of >60% (*t*_(13)_ = 2.87, *p* = 0.0066, one-tailed *t* test *n* = 5 images/condition; Fig. 3*D-F*). Such observations suggest that cLTP causes rapid depletion of ATF4 protein that starts in neuronal processes and is followed later by a decrease in the cell body.

### cLTP-induced ATF4 downregulation is mediated by ionotropic NMDA receptors

**Figure 4.**
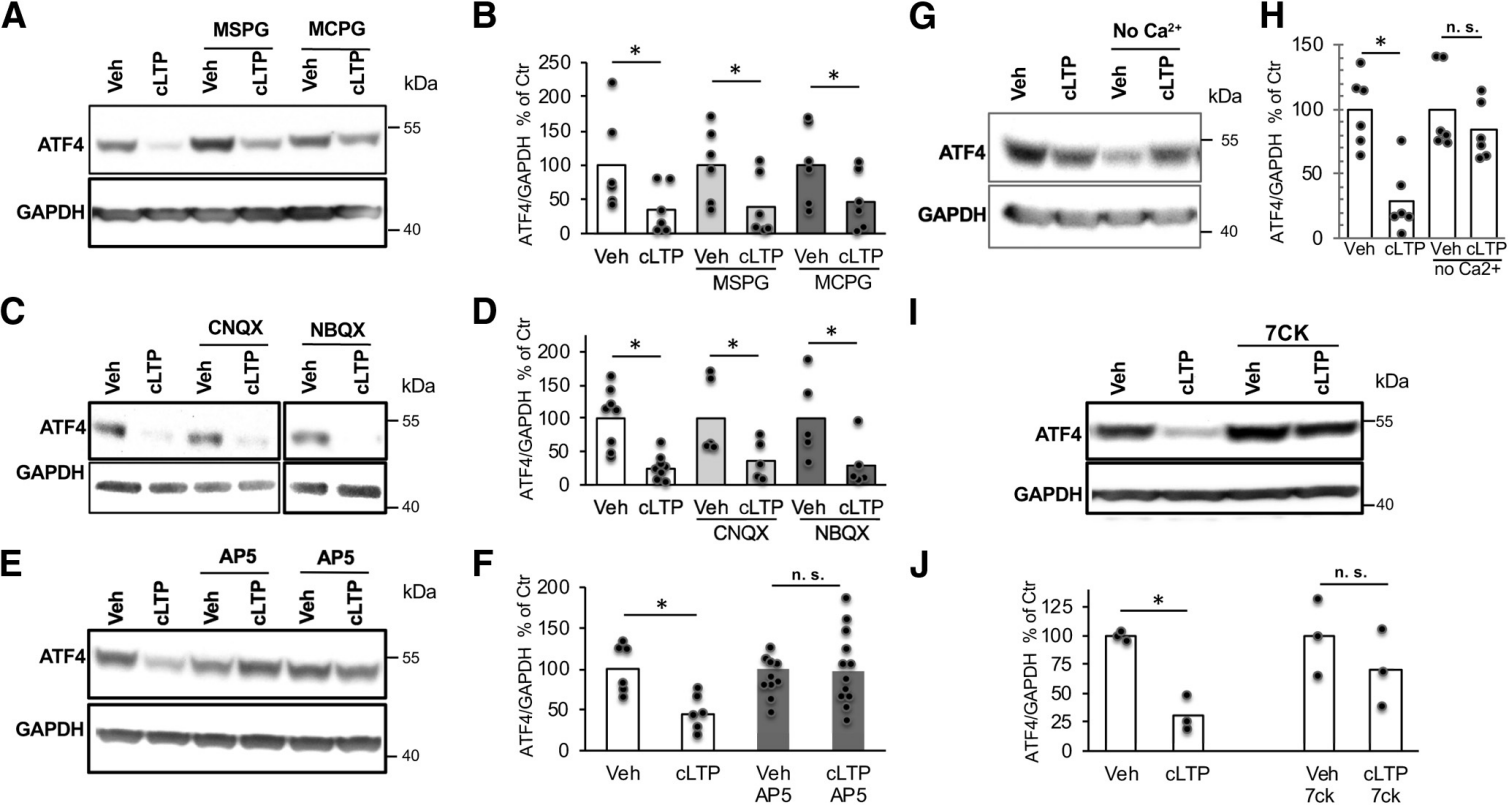
cLTP-induced ATF4 downregulation is dependent on NMDAR activation. ***A***, Representative WB of ATF4 and GAPDH in DIV 21 hippocampal cultures following pretreatment with metabotropic glutamate receptor antagonists (MCPG and MSPG) before cLTP induction or vehicle treatment. Protein was extracted at 1 h post-cLTP induction. ***B***, Quantification of multiple experiments as shown in ***A***. *N* = 6/condition. ***C***, Representative WB of ATF4 and GAPDH in DIV 21 hippocampal cultures following pretreatment with AMPA and kainate receptors antagonists (CNQX and NBQX) before cLTP induction or vehicle treatment. Protein was extracted at 1 h post-cLTP induction. ***D***, Quantification of multiple experiments as shown in ***C***. *N* = 5–8/condition. ***E***, Representative WB of ATF4 and GAPDH in DIV 21 hippocampal cultures following pretreatment with the NMDA receptor competitive antagonist (AP5) before cLTP induction or vehicle treatment. Protein was extracted at 1 h post-cLTP induction. ***F***, Quantification of multiple experiments as shown in ***E***. *N* = 5–12. ***G***, Representative WB of ATF4 and GAPDH in DIV 21 hippocampal cultures at 1 h following cLTP induction or vehicle treatment in the absence of Ca^2+^. ***H***, Quantification of multiple experiments as shown in ***G***. *N* = 6/condition**. *I***, Representative WB of ATF4 and GAPDH in DIV 21 hippocampal cultures following pretreatment with the antagonist 7CK for the ionotropic response of the NMDA receptor before cLTP induction or vehicle treatment. Protein was extracted at 1 h post-cLTP induction. ***J***, Quantification of ***I***. *N* = 3/condition.

Given that the cLTP protocol involves stimulation with glutamate, we examined which glutamate receptor mediates the rapid downregulation of ATF4. To achieve this, cultures were treated with antagonists for various types of ionotropic and metabotropic glutamate receptors before (15 min) and during cLTP induction, and then assessed for ATF4 levels 1 h following 30 s exposure to cLTP conditions. Treatment with MSPG [(±)-alpha-methyl-4-sulfonophenylglycine (group I and II metabotropic glutamate receptor antagonist)] or MCPG [(±)-alpha-methyl-4-carboxyphenylglycine (group II and III metabotropic glutamate receptor antagonist)] failed to block cLTP-induced ATF4 depletion (Fig. 4*A*,*B*; *t*_(10)_ = 1.91, *p* = 0.042, vehicle vs cLTP; *t*_(10)_ = 2.02, *p* = 0.035, MSPG vs MSPG+cLTP; *t*_(10)_ = 1.81, *p* = 0.05, MCPG vs MCPG+cLTP; all one-tailed *t* tests). Application of CNQX and NBQX (antagonists for ionotropic AMPA-responsive and kainate-responsive glutamate receptors) also did not prevent cLTP-induced ATF4 downregulation (Fig. 4*C*,*D*; *t*_(14)_ = 4.24, *p* = 0.0004, vehicle vs cLTP; *t*_(8)_ = 2.11, *p* = 0.034, CNQX vs CNQX+cLTP; *t*_(8)_ = 2.13, *p* = 0.033 for NBQX vs NBQX+cLTP; all one-tailed *t* tests). In contrast, pretreatment with the competitive NMDA receptor antagonist AP5 completely prevented the decrease in ATF4 protein levels promoted by cLTP (Fig. 4*E*,*F*; *t*_(10)_ = 3.47, *p* = 0.003 for vehicle vs cLTP; *t*_(21)_ = 0.141, *p* = 0.44 for AP5 vs AP5+cLTP; all one-tailed *t* tests). Another competitive NMDA receptor antagonist, 7CK, which binds to the glycine site of the receptor and antagonizes its ionotropic function without affecting metabotropic functions (Kemp et al., 1988; Nabavi et al., 2013) also prevented the cLTP-induced decrease in ATF4 protein levels (Fig. 4*I*,*J*; *t*_(4)_ = 7.4, *p* = 0.00089 for vehicle vs cLTP; *t*_(4)_ = 1.05, *p* = 0.19 for 7CK vs 7CK+cLTP; one-tailed *t* tests). Consistent with a role of ionotropic NMDA receptors in the downregulation of ATF4 following cLTP induction, we found that removing calcium (Ca^2+^) from the buffer during exposure to cLTP conditions prevented ATF4 downregulation (Fig. 4*G*,*H*; *t*_(10)_ = 4.5, *p* = 0.00057 vehicle vs cLTP; *t*_(10)_ = 0.99, *p* = 0.17 for no Ca^2+^ vs no Ca^2+^+cLTP, one-tailed *t* test). Altogether, these findings indicate that cLTP-dependent ATF4 downregulation requires NMDA receptor activation and consequent Ca^2+^ influx.

### ATF4 downregulation following cLTP induction appears in parallel with eIF2a dephosphorylation

**Figure 5.**
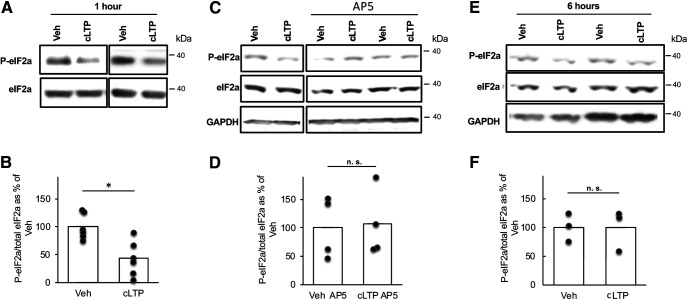
NMDAR-dependent decreased eIF2a phosphorylation following cLTP induction. ***A***, Representative WB of p-eIF2a and total eIF2a in DIV 21 hippocampal cultures 1 h following cLTP induction or vehicle treatment. ***B***, Quantification of multiple experiments as shown in ***A***. *N* = 6/condition. ***C***, Representative WB of p-eIF2a, total eiF2a, and GAPDH in DIV 21 hippocampal cultures at 1 h following cLTP induction or vehicle treatment after pretreatment without or with AP5 (NMDAR antagonist) as indicated. ***D***, Quantification of multiple experiments as shown in ***C***. *N* = 4/condition. ***E***, Representative WB of p-eIF2a, total eIF2a, and GAPDH in DIV 21 hippocampal cultures 6 h following cLTP induction or vehicle treatment. ***F***, Quantification of multiple experiments as shown in ***E***. *N* = 3/condition.

The absence of corresponding changes in ATF4 mRNA levels and the rapid local depletion of ATF4 protein in neuronal processes following cLTP induction suggest a nontranscriptional mechanism for cLTP regulation of ATF4 protein levels. Past work (Vattem and Wek, 2004) has established that ATF4 protein levels can be rapidly modulated at the level of translation in a variety of cell types, including neurons, by manipulating eIF2a. When phosphorylated at Ser52 in the rat, eIF2a promotes selective translation of a subset of transcripts, including that encoding ATF4; in contrast, ATF4 transcripts undergo highly inefficient translation in the presence of eIF2a not phosphorylated at Ser52. Of particular relevance, past work has shown that electrical stimulation that induces LTP in brain slices results in reduced eIF2a phosphorylation (Costa-Mattioli et al., 2007; Trinh et al., 2014), as do certain types of behavioral training (Jian et al., 2014; Werner et al., 2018). Therefore, we asked whether eIF2a phosphorylation is modulated following cLTP induction. eIF2a phosphorylation levels were significantly decreased 1 h following cLTP induction compared with the vehicle (Fig. 5*A*,*B*; *t*_(10)_ = 3.50, *p* = 0.0058, two-tailed *t* test). As in the case of ATF4 depletion, inhibiting NMDARs with AP5 blocked cLTP-induced p-eIF2a downregulation (Fig. 5*C*,*D*; *t*_(6)_ = 0.16, *p* = 0.88, two-tailed *t* test). Similar to the dynamic changes in ATF4 protein levels, p-eIF2a levels returned to baseline (*t*_(4)_ = 0.0048, *p* = 1, two-tailed *t* test) at 6 h following cLTP induction (Fig. 5*E*,*F*). These observations suggest that under our experimental conditions, cLTP causes a transient NMDA receptor-dependent decrease in eIF2a phosphorylation that in turn correlates with the time-dependent changes in neuronal ATF4 expression.

### ATF4 downregulation is required for resetting the increased density of synaptic AMPA receptors induced by cLTP back to baseline

**Figure 6.**
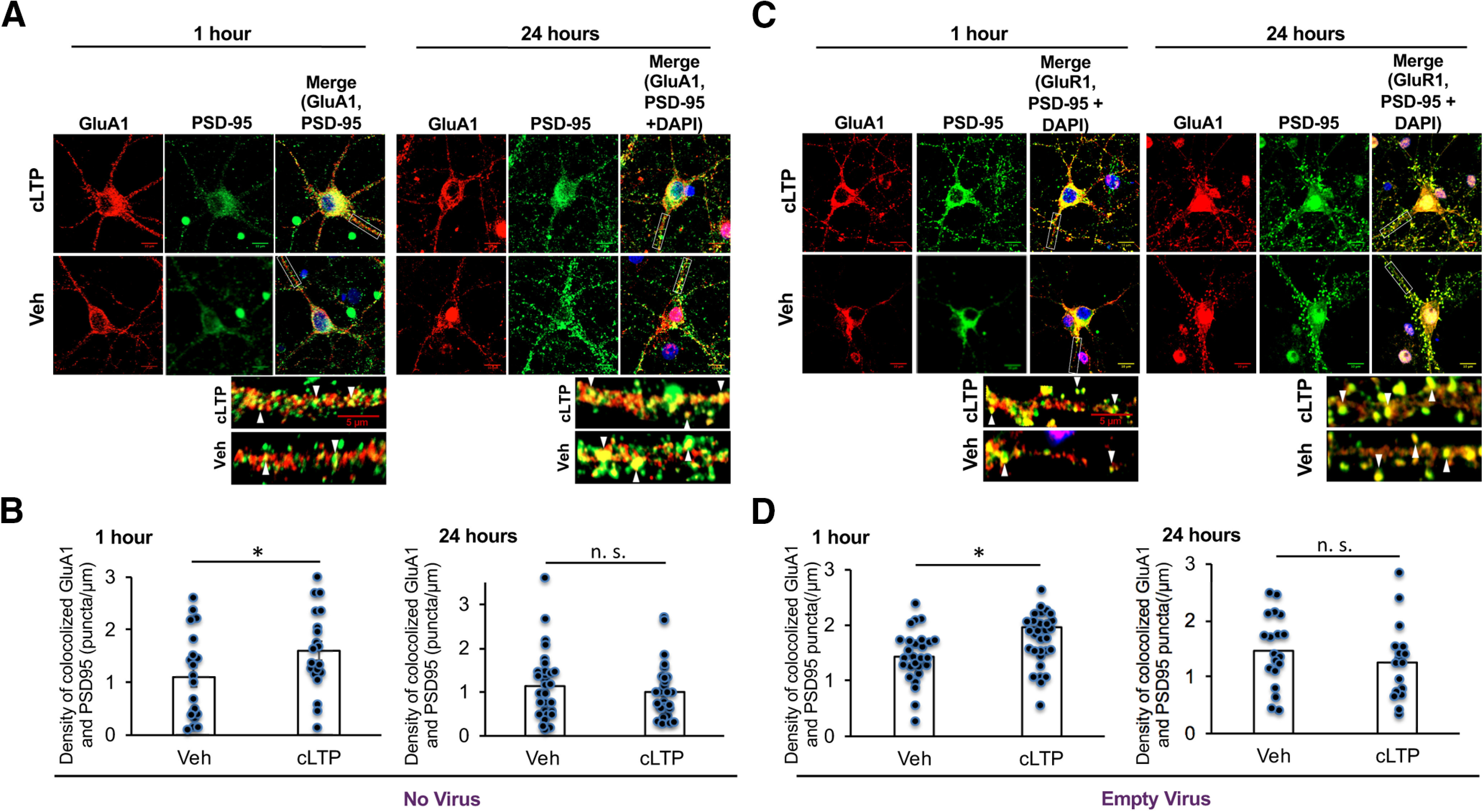
Synaptic GluA1/PSD puncta density increases at 1 h and returns back to baseline at 24 h following cLTP, while infection with empty lentivirus has no effect on cLTP induction or resetting of GluA1/PSD-95 puncta density. ***A***, Immunofluorescent labeling at 1 h post-cLTP or vehicle treatment for surface GluA1 (AF568, red), PSD-95 (AF680, green), and DAPI (blue) in DIV21 hippocampal cultures. Colocalized GluA1/PSD-95 puncta (yellow) correspond to postsynaptically located AMPARs. ***B***, Quantification of images as in ***A***, as puncta per micrometer, at 1 h (*N* = 20/condition) or 24 h (*N* = 29/condition). ***C***, Immunofluorescent labeling at 1 or 24 h post-cLTP induction or vehicle treatment for surface GluA1 (AF568, red), PSD-95 (AF680, green), and DAPI (blue) in cultures of 21 DIV hippocampal neurons infected with empty lentivirus. Colocalized GluA1/PSD-95 puncta (yellow) correspond to postsynaptically located AMPARs. Viral treatment was 24 h before cLTP induction or treatment with vehicle. ***D***, Quantification of images as in ***C***. *N* = 28–30/condition at 1 h and 17–18/condition at 24 h. Scale bars: 10 μm; insets, 5 μm.

Synaptic plasticity is characterized by molecular and structural alterations that account for changes in synaptic efficacy. These include delivery of ionotropic glutamatergic AMPA receptors (AMPARs) from extrasynaptic sites and/or from internal stores to silent synapses (Lu et al., 2001; Park et al., 2004), thus converting them to an active state that promotes LTP (Liao et al., 1995; Durand et al., 1996), as well as the remodeling of dendritic spines (Bosch et al., 2014). Additionally, although LTP can persist for at least several hours (Villers et al., 2014), it must be reversible so that synapses can be reset for additional rounds of potentiation (Abraham and Bear, 1996). In this context, we examined whether cLTP induction promotes time-dependent changes in AMPAR localization at postsynaptic densities in cultured hippocampal neurons and whether such changes might be affected by levels of ATF4. We first immunofluorescently labeled surface GluA1 (AMPAR subunit) and PSD-95 (a marker of postsynaptic densities) on dendrites of mature cultured hippocampal neurons to determine whether cLTP induces changes in the density of synapse-associated GluA1/PSD-95 puncta. At 1 h following cLTP induction, the density of GluA1/PSD-95 puncta was significantly elevated (*t*_(38)_ = 1.72, *p*  =  0.049, one-tailed *t* test) compared with vehicle control; Fig. 6*A*, left, *B*, left). These observations are consistent with previous studies showing elevation of synaptic AMPAR density at up to 3 h post-LTP induction (Liao et al., 1995; Malenka and Nicoll, 1999; Bredt and Nicoll, 2003). In contrast, when we examined GluA1/PSD-95 puncta density at 24 h post-cLTP initiation, this parameter was no longer elevated compared with vehicle controls (*t*_(56)_ = 0.54, *p* = 0.30, one-tailed *t* test; Fig. 6*A*, right, *B*, right), indicating that the synaptic AMPAR density had “reset” to baseline by this time.

**Figure 7.**
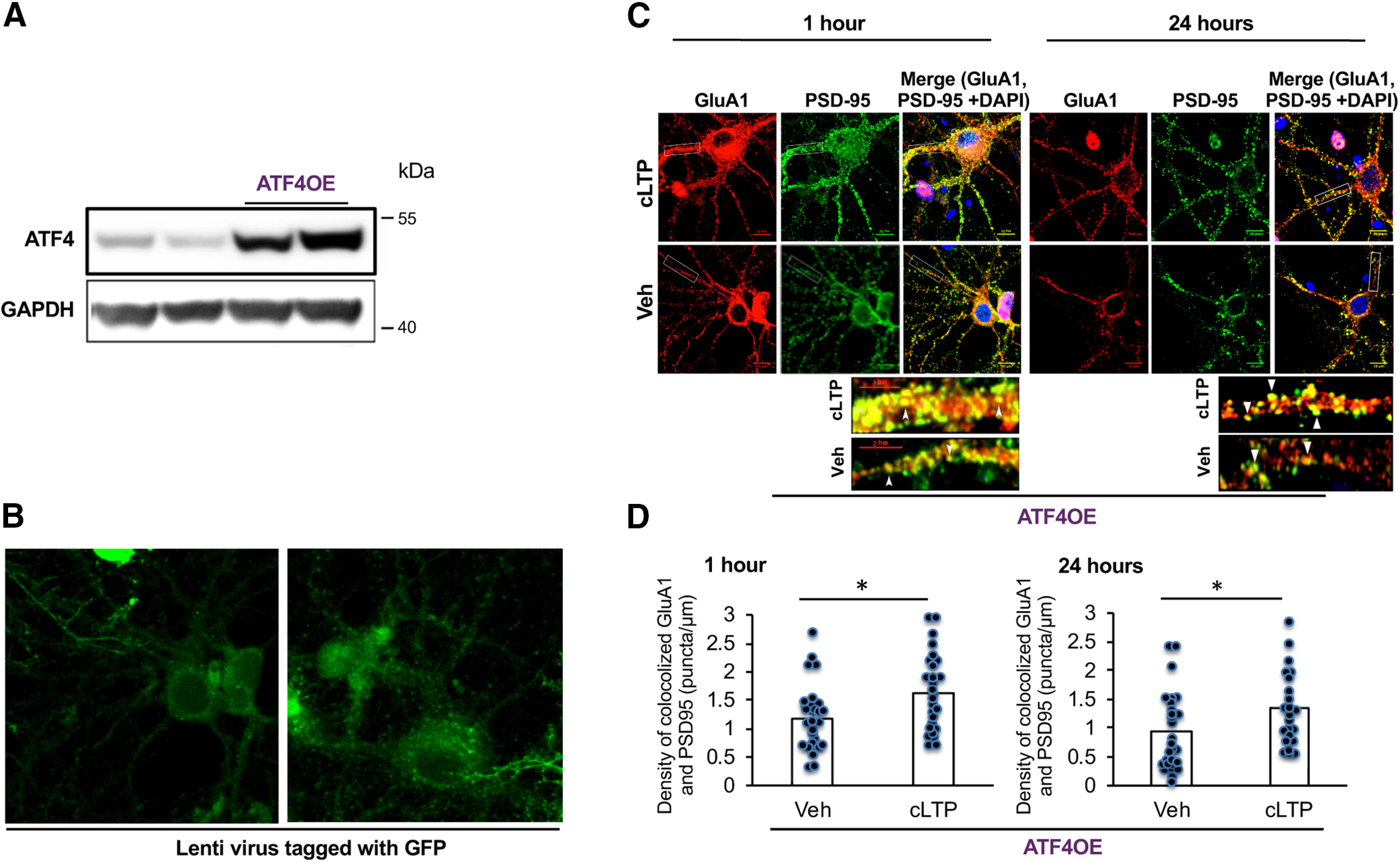
Overexpression of ATF4 protein prevents the return of synaptic GluA1/PSD puncta density back to baseline at 24 h following cLTP. ***A***, Representative WB of ATF4 and GAPDH in hippocampal cultures infected with ATF4 overexpressing lentivirus for 24 h. ***B***, Immunofluorescent labeling of GFP in hippocampal cultures infected with lentivirus expressing both GFP and ATF4 confirms successful infection. ***C***, Immunofluorescent labeling at 1 or 24 h post-cLTP or vehicle treatment for surface GluA1 (AF568, red), PSD-95 (AF680, green), and DAPI (blue) in ATF4-overexpressing DIV21 hippocampal cultures. ***D***, Quantification of images as in ***C***, as puncta per micrometer. *N* = 29–30/condition at 1 h and 22–27/condition at 24 h. ATF4OE, ATF4 overexpression. Scale bars: 10 μm; inset, 5 μm.

To evaluate whether ATF4 downregulation caused by cLTP plays a role in the return of the density of GluA1/PSD-95 puncta to baseline, we examined the density of puncta in cultures in which ATF4 was overexpressed (Fig. 7*A*,*B*). Our past work established that ATF4 overexpression does not affect baseline levels of puncta density (Liu et al., 2014), a finding that we verified in the present study (Fig. 7*C*,*D*). Moreover, ATF4 overexpression did not alter the occurrence or magnitude of the elevation in GluA1/PSD-95 puncta density induced by cLTP (*t*_(57)_ = 3.24, *p* = 0.001, one-tailed *t* test; Fig. 7*C*, left, *D*, left). Significantly, at 24 h post-cLTP, GluA1/PSD-95 puncta density in ATF4-overexpressing cultures remained elevated (*t*_(47)_ = 1.88, *p* = 0.033, one-tailed *t* test) at the levels seen at 1 h, and, in contrast with control cultures (Fig. 6), did not return to baseline (Fig. 7*C*, right, *D*, right). These effects were not because of the transduction protocol since infection with empty virus had no effect on cLTP-induced GluA1/PSD-95 puncta increase at 1 h (*t*_(56)_ = 2.41, *p* = 0.0099, one-tailed *t* test) or return to baseline at 24 h (*t*_(33)_ = 0.9, *p* = 0.19, one-tailed *t* test) post-cLTP induction (Fig. 6*C*,*D*). Together, these findings indicate that the elevation of synaptic AMPAR density that occurs in response to, and underlies, cLTP initiation returns to baseline within 24 h and that this resetting is dependent on a transient decrease in ATF4 levels.

### cLTP-induced ATF4 downregulation is required for resetting synaptic potentiation back to baseline

**Figure 8.**
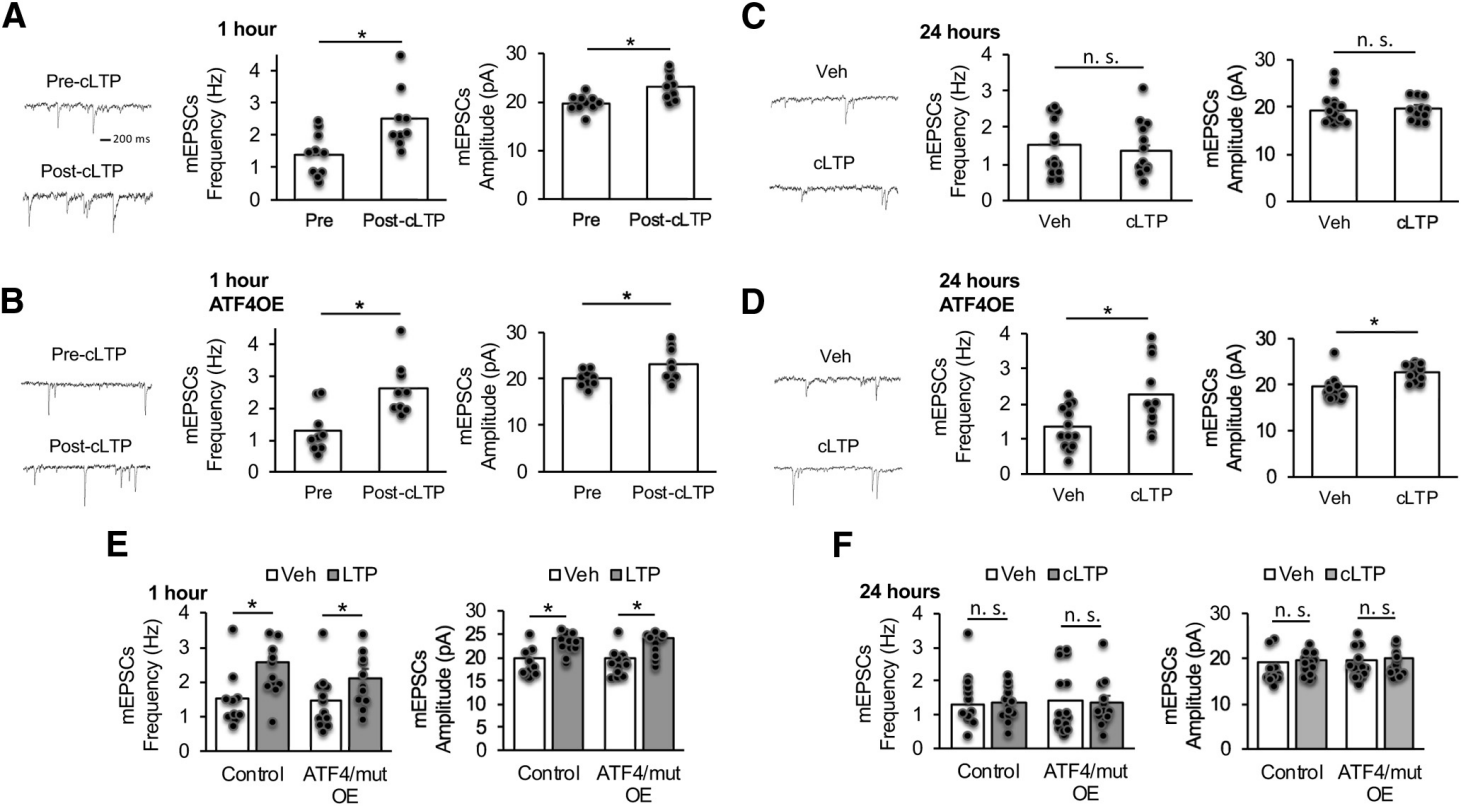
cLTP-dependent ATF4 downregulation is required for resetting cLTP-induced synaptic potentiation back to baseline. ***A***, Left, Representative mEPSC traces recorded from 21 DIV hippocampal neurons before or 1 h after cLTP induction. Right, Bar graphs represent the frequency (left) and the amplitude (right) of mEPSCs (*N* = 9–10/condition). ***B***, Left, Representative mEPSC traces recorded from 21 DIV hippocampal neurons infected for 24 h with ATF4-expressing lentivirus before and 1 h after cLTP induction. Right, Bar graphs represent the frequency (left) and the amplitude (right) of mEPSCs (*N* = 9/condition). ***C***, Left, Representative mEPSC traces recorded from 21 DIV hippocampal neurons 24 h after cLTP induction or vehicle treatment. Right, Bar graphs represent the frequency (left) and the amplitude (right) of mEPSCs (*N* = 12–14/condition). ***D***, Left, Representative mEPSC traces recorded from 21 DIV hippocampal neurons infected for 24 h with ATF4 expressing lentivirus and 24 h after cLTP induction or vehicle treatment. Right, Bar graphs represent the frequency (left) and the amplitude (right) of mEPSCs (*N* = 12–13/condition). ***E***, Frequency (left) and amplitude (right) of mEPSCs recorded from 21 DIV hippocampal neurons infected for 24 h with control or ATF4 transcriptionally inactive expressing lentivirus before and 1 h after cLTP induction or vehicle treatment. *N* = 10 for control virus; *N* = 11 for ATF4/mut OE virus. ***F***, Frequency (left) and amplitude (right) of mEPSCs recorded from 21 DIV hippocampal neurons infected for 24 h with control or ATF4 transcriptionally inactive expressing lentivirus 24 h after cLTP induction or vehicle treatment (*N* = 11–16/condition). ATF4OE, ATF4 overexpression; ATF4/mut OE, overexpression of transcriptionally inactive ATF4.

The observation that transient ATF4 depletion appears to be required for resetting the synaptic AMPAR density by 24 h after cLTP suggests that the loss of ATF4 may also play an obligate role in resetting the electrophysiological manifestations of cLTP. To assess this, we compared mEPSC frequency and amplitude at 1 and 24 h post-cLTP in mature hippocampal cultures with or without ATF4 overexpression. Our past studies established that ATF4 overexpression alone does not alter these parameters (Pasini, et al., 2015). As seen at 30 min following cLTP (Fig. 1*A*), both mEPSC frequency (*t*_(17)_ = 2.82, *p* = 0.006, one-tailed *t* test) and amplitude (*t*_(17)_ = 3.23, *p* = 0.0025, one-tailed *t* test) were significantly elevated at 1 h post-cLTP in control cultures without ATF4 overexpression (Fig. 8*A*). Moreover, ATF4 overexpression had no effect on changes in frequency (*t*_(16)_ = 3.46, *p* = 0.0016, one-tailed *t* test) or amplitude (*t*_(16)_ = 2.15, *p* = 0.024, one-tailed *t* test) induced by cLTP comparing post- to pre-cLTP (Fig. 8*B*). Additionally, baseline values for mEPSC frequency and amplitude in cultures before cLTP induction were no different in neurons with ATF4 overexpression compared with control cultures (Fig. 8*A*,*B*). Consistent with the resetting observed for GluA1/PSD-95 puncta density at 24 h post-cLTP, both mESPC frequency (*t*_(24)_ = 0.59, *p* = 0.48, one-tailed *t* test) and amplitude (*t*_(24)_ = 0.21, *p* = 0.42, one-tailed *t* test) returned to baseline by this time post-cLTP, and these values were no different compared with those of neurons treated with vehicle (Fig. 8*C*). In contrast, with ATF4 overexpression, mEPSC frequency (*t*_(23)_ = 2.95, *p* = 0.0035, one-tailed *t* test) and amplitude (*t*_(23)_ = 3.63, *p* = 0.00065, one-tailed *t* test) remained significantly elevated at 24 h post-cLTP compared with vehicle (Fig. 8*D*). At this time, again ATF4 overexpression alone had no effect on mEPSC frequency or amplitude compared with controls (Fig. 8*C*,*D*).

Given that ATF4 is a transcription factor, we posed the additional question of whether the effects of ATF4 overexpression on cLTP prolongation requires its transcriptional role. To address this, we overexpressed a mutant ATF4 construct, ATF4add/mut, that encodes a mutated ATF4 that does not bind DNA, and that thus is transcriptionally inactive (Liu et al., 2014; Corona et al., 2018). Overexpression of this construct does not phenocopy the actions of ATF4 knockdown on synaptic density, and hence it does not appear to have dominant-negative properties that might mimic ATF4 depletion (Liu et al., 2014). Similar to wild-type ATF4 overexpression, ATF4add/mut overexpression did not affect the initiation phase of cLTP, as indicated by the elevation of mEPSC frequency (*t*_(19)_ = 2.4, *p* = 0.014 for control virus; *t*_(21)_ = 1.79, *p* = 0.044 for ATF4add/mut virus; one-tailed *t* test) and amplitude (*t*_(19)_ = 3.4, *p* = 0.0015, for control virus; *t*_(21)_ = 3.59, *p* = 0.00085 for ATF4add/mut virus; one-tailed *t* tests). (Fig. 8*E*). However, in contrast to overexpressed wild-type ATF4 (Fig. 8*D*), overexpressing ATF4add/mut virus did not prevent the resetting of the mESPC frequency (*t*_(23)_ = 0.54, *p* = 0.29 for control virus; *t*_(27)_ = 0.3, *p* = 0.39 for ATF4add/mut virus; one-tailed *t* test) or amplitude (*t*_(22)_ = 0.29, *p* = 0.39 for control virus; *t*_(27)_ = 0.32, *p* = 0.38 for ATF4add/mut virus; one-tailed *t* test) back to baseline at 24 h following cLTP induction compared with vehicle (Fig. 8*F*). Together, these findings support a role for ATF4 downregulation in returning the potentiation state of hippocampal neurons back to baseline following cLTP and for a transcriptional role of ATF4 in this regard.

## Discussion

The present study addresses two outstanding questions regarding the role of ATF4 in neuronal plasticity. First, are there physiologic conditions under which ATF4 levels are modulated? Second, if so, what are the consequences of such modulation? Here, we report that ATF4 protein expression rapidly diminishes in response to cLTP induced by NMDA receptor stimulation and that this loss of ATF4 is required for the decay to baseline of synaptic excitatory receptor density and of potentiation that occur under our experimental conditions within 24 h. As discussed further, these observations are consistent with the idea that ATF4 functions as a feedback regulator to ensure the control of neuronal plasticity.

Numerous studies have documented that cellular stress can increase ATF4 protein levels via increases in both ATF4 mRNA and p-eIF2a-dependent translation (Vattem and Wek, 2004; Dey et al., 2010; Baleriola et al., 2014; Mazor and Stipanuk, 2016), and that elevated ATF4 levels can promote either cell death or survival depending on the context. Because exposure to excessive glutamate can cause neuronal degeneration and death (Olney, 1986; Meldrum and Garthwaite, 1990; Choi, 1992), we considered the possibility that our cLTP conditions (50 μm glutamate for 30 s) may cause excitotoxicity that could underlie our observed changes in ATF4 protein levels. However, we saw no evidence of neuronal loss or degeneration under the conditions of our studies, nor did we detect activation of caspase 3. This is consistent with reports that brief exposure to low levels of glutamate or NMDA do not cause toxicity (Hardingham, 2009). Moreover, we did not see elevations in p-eIF2a or ATF4 indicative of a stress response, but rather a decrease in both parameters. The effects of cLTP on ATF4 levels were also very rapid, occurring within 15 min of treatment, which is well before any potential toxic responses.

Our pharmacologic studies indicate that the first steps in the reduction of ATF4 under cLTP conditions are excitation of NMDAR and inflow of Ca^2+^. This appears to be followed by a decrease in phosphorylation of eIF2a, which in turn leads to reduced translation of ATF4 transcripts. Past work has established that LTP conditions can diminish eIF2a phosphorylation (Costa-Mattioli et al., 2007). It is presently unclear whether such an effect reflects increased p-eiF2a dephosphorylation or decreased kinase-dependent eiF2a phosphorylation activity, and it will be important in future studies to dissect the steps that lie between NMDAR activation, Ca^2+^ entry, and changes in eIF2a phosphorylation state. The fall in ATF4 that occurs in response to cLTP is rapid and detectable in processes within 15 min by immunostaining, but not in the cell body where the effect is detectable only by 1 h. This suggests that the initial ATF4 response is in dendrites and that this is later reflected in what is transported to the cell body and nucleus. The plausibility of this scenario is supported by observations that ATF4 can be retrogradely transported from distal processes to neuronal nuclei (Lai et al., 2008).

Establishment and maintenance of LTP is characterized by increased sensitivity to synaptic stimulation and is associated with increased excitatory glutamate receptor trafficking to the neuronal surface and enhanced postsynaptic excitatory receptor density (Herring and Nicoll, 2016). In line with this, we observed significant increases in mEPSC frequency and amplitude at 30 min to 1 h post-cLTP and found an elevated density of GluA1/PSD-95 puncta at the latter time. Although LTP can be durable, it can also decay depending on the conditions of stimulus and preparation. In our cultures, both mESPC responses and excitatory receptor density returned to pre-cLTP baseline levels by 24 h after treatment. Given our previously described roles of ATF4 in receptor trafficking and excitatory synapse formation, we tested the hypothesis that the “resetting” of mEPSC responses and GluA1/PSD-95 puncta density seen at 24 h post-cLTP would depend on the depletion of ATF4. In support, the reset to baseline was reversed by ATF4 overexpression so that mEPSC frequency and amplitude as well as GluA1/PSD-95 density remained elevated 24 h after cLTP. Moreover, these actions of ATF4 overexpression were not reproduced by an ATF4 mutant lacking transcriptional activity, thus indicating a transcription-dependent mechanism. Of note, the absence of cLTP resetting at 24 h observed with ATF4 overexpression was not because of ATF4-dependent stimulation of the cLTP response, as no such effect was seen at 1 h post-cLTP.

Because ATF4 is a transcription factor, it will be important in the future to query the extent to which the cLTP-promoted decline in ATF4 levels is reflected in altered gene expression and how such changes might account for the association between reduction in ATF4 levels and resetting of mEPSC responses and excitatory synapse density to baseline at 24 h post-cLTP. We have reported a number of changes in gene expression that occur in response to ATF4 downregulation in hippocampal cultures and that could be relevant to the current observations (Pasini et al., 2016; Liu et al., 2018). Though presently speculative, one intriguing possibility is via Arhgdia (encoding RhoGDIa), a direct, positively regulated ATF4 target (Pasini et al., 2016). RhoGDIa acts as a major controller of the activity, localization, and stability of Rho family GTPases. We have reported that neuronal ATF4 depletion destabilizes the Rho GTPase family member Cdc42 via the downregulation of RhoGDIa (Liu et al., 2014; Pasini et al., 2016). Loss of Cdc42 in neurons affects synaptic plasticity with decreased density of dendritic spines and excitatory synapses (Kim et al., 2014; Liu et al., 2014) as well as altered surface expression of AMPAR (Hussain et al., 2015). Thus, one possible component of the pathway downstream of cLTP-dependent ATF4 depletion that could contribute to resetting of potentiation is diminished transcription of Arhgdia followed by reduction of Cdc42 levels and consequent decline in GluA1-containing synapses.

The model we used here, cLTP in mature hippocampal cultures, facilitated the measurement of biochemical and structural responses to NMDAR-promoted potentiation as well as facile manipulation of ATF4 expression. This raises the question of whether our findings pertain to learning in intact animals or to other forms of LTP and in particular those observed in brain slices or in the intact brain. In this regard, p-eIF2a levels are reported to decrease within 15–30 min of contextual fear conditioning in rat dorsal hippocampus (Costa-Mattioli et al., 2007) and in rat gustatory cortex within 5 min of novel taste learning (Stern et al., 2013). p-eIF2a levels were also shown to fall in mouse hippocampal slices within 15 min of a strong LTP stimulus or within 5 min of a forskolin-promoted cLTP protocol (Costa-Mattioli et al., 2005). Finally, the subjection of mouse hippocampal slices to a forskolin-rolipram-induced cLTP reduced ATF4 protein levels within 15–30 min, although in this case, the reduction was ascribed to elevated ATF4 proteasomal degradation (Dong et al., 2008).

**Figure 9.**
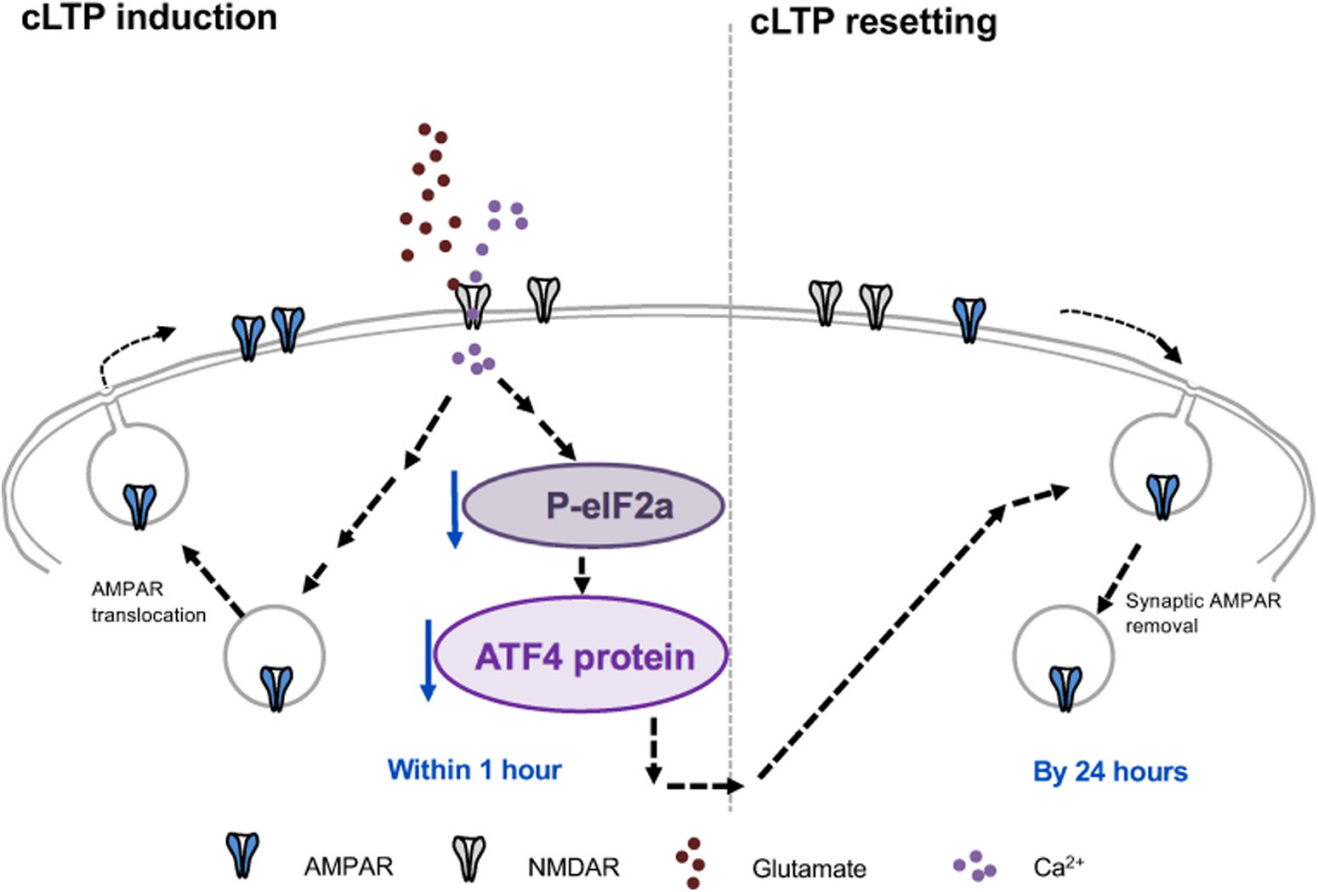
Model for role of ATF4 regulation in resetting of excitatory synapse density and potentiation in response to cLTP. Glutamate-induced chemical LTP leads to a rapid downregulation of ATF4 protein levels via decreased phosphorylation of eIF2a and consequent reduction in ATF4 mRNA translation. The resulting ATF4 protein decrease is required for active resetting of synaptic AMPARs density, thereby returning synaptic responses to baseline levels to prevent saturation and to make synapses more responsive to future stimuli.

Together, our findings suggest a feedback mechanism of the following chain of linked events: (1) rapid reduction of eIF2a phosphorylation in response to NMDAR-dependent LTP; (2) decreased translation of ATF4 mRNA and consequent ATF4 protein depletion in dendrites; (3) delayed reduction of ATF4 levels in cell bodies; (4) altered expression of ATF4-regulated genes; (5) reduction of excitatory synaptic receptor density (as previously seen with ATF4 downregulation; Liu et al., 2014); and (6) resetting of stimulation responsiveness to baseline, pre-LTP conditions (Fig. 9). What might be the potential physiologic relevance of such a mechanism? An appealing possibility is as a contributor to synaptic regulation. Within this framework, one role of LTP-dependent ATF4 depletion may be to reset synapses so that LTP “saturation” (Moser et al., 1998) is avoided and responsiveness to potentiation is restored, thereby permitting additional rounds of plasticity in response to a changing environment. Such a feedback mechanism could additionally act to provide regulation so that circuits do not undergo uncontrolled and potentially catastrophic feedforward potentiation (McEachern and Shaw, 1999; Bliss and Cooke, 2011). Finally, the mechanisms described here may play a part in synaptic “scaling,” in which there is a weighted, global reduction in synaptic strength at all synapses in a given neuron in response to activity (Turrigiano, 2008). A role for ATF4 as a feedback regulator of neuronal excitability would be in keeping with our current understanding of ATF4 as a rapid response and control element in pathways designed to maintain and restore cellular homeostasis (Baird and Wek, 2012; Pakos-Zebrucka et al., 2016).

## Synthesis

Reviewing Editor: Katalin Toth, University of Ottawa Decisions are customarily a result of the Reviewing Editor and the peer reviewers coming together and discussing their recommendations until a consensus is reached. When revisions are invited, a fact-based synthesis statement explaining their decision and outlining what is needed to prepare a revision will be listed below. The following reviewer(s) agreed to reveal their identity: Suzanne Paradis. Note: If this manuscript was transferred from JNeurosci and a decision was made to accept the manuscript without peer review, a brief statement to this effect will instead be what is listed below.

The study establishes a correlation between ATF4 protein levels, phospho-eIf2a levels, and mEPSC amplitude “resetting” in response to cLTP. The study provides useful information, however the antibody specificity should be clearly demonstrated as outlined by Reviewer #2. This control experiments would significantly increase the strength of the authors’ conclusion.

Rev.#1.

The authors have addressed my comments.

I have some minor points the authors must consider.

1) The phosphorylation site on eIF2 is Ser51 not Ser52.

2) “Past work has shown that electrical stimulation that induces LTP in brain slices results in reduced eIF2a phosphorylation (Costa-Mattioli et al., 2007)” should also include (Trinh et al., Learn Mem, 2014).

3) Behavioral training that reduces eIF2 phosphorylation should also include (Jian et al., JN, 2014; Werner et al., JN, 2018; Eacker et al., bioRxiv, 2017).

Rev. #2.

1) The authors failed to demonstrate that their ATF4 antibody specifically detects ATF4 in fixed tissue. The immunostained images presented in their rebuttal are at too low of a magnification and are poorly described (e.g. what age of animal?) to be convincing; no quantification of staining is provided. The relevant control experiment that the authors should provide is to culture neurons from the ATF4 KO mouse and quantify antibody staining in dendrites and soma as in Fig. 3. If the authors were to perform this careful analysis it should be included in the main body of the paper.

2) The model presented by the authors remains opaque. The authors fail to supply data supporting a model whereby ATF4 is retrogradely transported from synapse to nucleus to regulate gene expression. (There is one paper that the authors cite to support this assertion from 2008 and not from their own lab). Further, where does eIF2a fit into the picture (e.g. is it also at the synapse and being transported)? In fact, their own model Fig. 9 is vague about subcellular localization of the relevant proteins.

3) The revised version of this manuscript fails to increase the overall rigor of the study, which was lacking in the original submission, also.
