# Supplementary figures and images for: Rapid ATF4 Depletion Resets Synaptic Responsiveness after cLTP
Source: eNeuro. 2021 Jun 2;8(3):ENEURO.0239-20.2021. doi: 10.1523/ENEURO.0239-20.2021 (PMC8177969; doi:10.1523/ENEURO.0239-20.2021)

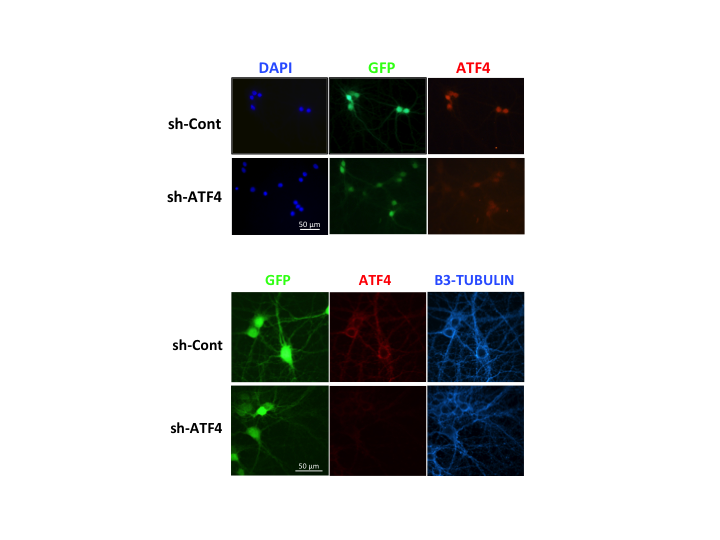

Supplement: Figure 3-1 — Validation of the antibody used for immunofluorescent staining of cultured hippocampal neurons. DIV 7 cultures were infected with lentivirus expressing either GFP (control) or GFP + shATF4, and fixed and stained on DIV 21. For ATF4 labeling, following fixing, cells were probed as described in Materials and Methods with ATF4 antibody (1:300; Cell Signaling Technology), rabbit monoclonal antibody D4B8 (catalog #11815, Thermo Fisher Scientific), and then a secondary anti-rabbit antibody, Alexa Fluor 568 (1:500; Thermo Fisher Scientific) after permeabilization. For β-III-tubulin, labeling was conducted with mouse monoclonal antibody TU-20 (1:300; catalog #NB-600-1018, Novus Biologicals), then a secondary anti-mouse antibody, Alexa Fluor 647 (1:500; Thermo Fisher Scientific). Top, Staining as imaged on an upright fluorescent microscope. Bottom, Staining as imaged by confocal microscopy (merged z-stack). Download Figure 3-1, TIF file. [file enu-eN-NWR-0239-20-s01.tif]
